# Innovative Methods for Reducing Sodium in Cupcakes With Mechanical Leavening and Ozonized Water

**DOI:** 10.1155/ijfo/4706504

**Published:** 2024-12-10

**Authors:** Maxat N. Mamyrayev, Auyelbek Iztayev, Talgat K. Kulazhanov, Madina A. Yakiyayeva, Mira E. Yerzhanova, Erik B. Askarbekov, Bayan Zh. Muldabekova, Pernekul M. Maliktayeva, Sholpan A. Tursunbayeva

**Affiliations:** ^1^Department of Technology of Bread Products and Processing Industries, Almaty Technological University, Almaty, Kazakhstan; ^2^Research Institute of Food Technologies, Almaty Technological University, Almaty, Kazakhstan; ^3^Career and Business Partnership Center, M.Kh. Dulaty Taraz Regional University, Taraz, Kazakhstan; ^4^Department of Standardization and Veterinary Sanitation, International Taraz Innovation Institute, Taraz, Kazakhstan

**Keywords:** cupcake, fine whole-ground flour, mechanical loosening, ozonated water, technology

## Abstract

This study developed a technology to prepare cupcakes with a reduced sodium content from fine whole-ground wheat flour and ozonated water. Cavitation technology was utilised to reduce the sodium content, to shorten the cooking time, and to improve the consistency of the dough by accelerated churning. The dough was obtained by cavitation loosening under compressed air pressure and increasing the speed to 900 rpm. The cupcake preparation was accelerated by increasing the number of revolutions, resulting in a more saturated dough and a two-to-threefold volume increase. This approach produced a product of good quality and eliminated baking powder from the recipe. The optimal parameters for preparing dough using accelerated ozone cavitation technology were determined: an ozone concentration of 3 mg/cm^3^, 900 rpm of the kneading body, and a dough chamber pressure of 0.3 MPa. This study has established that accelerated ozone cavitation technology contributes to ensuring food safety, increasing production efficiency, and ensuring the quality of the finished product. This technology allows manufacturers to obtain high-quality and safe flour confectionery products using an accelerated method.

## 1. Introduction

In recent years, there has been growing attention on reducing sodium levels in food products due to the known health risks of excessive sodium intake, including hypertension, cardiovascular disease, and kidney damage. The food industry is actively seeking innovative methods to reduce sodium without compromising taste, texture, or safety. A promising approach involves the use of mechanical leavening agents combined with ozonized water to achieve desirable product characteristics while lowering sodium content [1, 2]. This paper examines the effects of mechanical leavening and ozonated water on the sodium content, textural properties, and sensory characteristics of cupcakes and highlights the promising potential of these methods to reduce sodium in baked goods.

Sodium is a naturally occurring element found in a variety of foods and plays a critical role in human biological processes such as maintaining electrolyte balance, nerve transmission, and muscle function. Sodium is also found in food additives such as baking powder and baking soda, which are often used as a leavening agent in desserts. However, excessive sodium intake is a major concern as it is associated with various cardiovascular diseases such as hypertension, heart failure, and stroke [3, 4]. Given that excessive sodium consumption can lead to numerous health complications, health organisations have become increasingly focused on sodium intake levels, advocating for a reduction in daily sodium intake. According to the World Health Organization (WHO), the recommended daily intake of sodium should not exceed 2000 mg, which is equivalent to 5 g of salt. Research has shown that in many countries, dietary sodium intake exceeds this recommended value, leading to health problems among the population.

High sodium intake is associated with a number of complications, which primarily affect cardiovascular health. There are notable health problems associated with excess sodium intake: (a) there is a well-documented link between high sodium intake and increased blood pressure (hypertension). High blood pressure can damage artery walls and strain the heart, increasing the risk of heart disease, stroke, and kidney disease. (b) Excessive sodium intake may contribute to the development of coronary heart disease. The buildup of plaque in the arteries due to high blood pressure narrows the arterial lumens, resulting in restricted blood flow to the heart. This can lead to chest pain, heart attacks, and even heart failure. (c) High sodium intake increases the risk of stroke because hypertension is a major risk factor for both ischaemic and haemorrhagic stroke. A stroke occurs when the blood supply to part of the brain is cut off, leading to cell death and potential long-term disability or death. (d) Chronically high blood pressure caused by excess sodium intake can damage the delicate blood vessels in the kidneys. This can impair kidney function and lead to decreased efficiency in eliminating waste and an inability to maintain fluid balance in the body. (e) High sodium intake can cause calcium to be excreted through the kidneys, which negatively affects bone mineral density and can lead to osteoporosis. Therefore, excessive sodium intake may contribute to the development of osteoporosis and increase the risk of bone fractures. The balance between healthy sodium intake and the ubiquity of sodium in the food industry, especially in desserts, requires innovative approaches to reduce sodium without compromising flavor and texture [5–7].

Mechanical leavening leverages the introduction of air into the dough, which creates a network of bubbles that expand during baking, leading to a light and airy structure. This process is enhanced by using ozonated water, which facilitates the oxidation of sulfhydryl groups in gluten proteins. The oxidation improves gluten cross-linking, contributing to the structural integrity of the final product without relying on traditional sodium-based leavening agents. By eliminating the need for baking powder and baking soda, this method effectively reduces the overall sodium content in the final product [8, 9]. Addressing this challenge requires balancing the key aspects of taste, texture, and food safety while reducing the sodium content [10, 11].

Several strategies have been proposed to reduce the sodium content in desserts, ranging from direct replacement of sodium-containing agents to the use of other processes that help increase the volume and texture of desserts. Mechanical leavening and ozonated water are innovative technologies that promise to reduce the amount of sodium in baked goods such as fruit pies and muffins [12, 13].

Replacing traditional sodium-containing leavening agents such as sodium bicarbonate and sodium aluminium phosphate with low-sodium alternatives such as potassium bicarbonate and monocalcium phosphate can effectively reduce the amount of sodium in desserts. However, such substitutions may affect flavor and texture, requiring changes to the recipe and ingredient balance [14–16].

The application of ozonated water in the food industry has been increasingly recognized for its potential to improve product safety and quality, particularly in bakery products. Several studies have highlighted the benefits of ozonated water in various food processing applications, including its ability to reduce microbial load, enhance shelf life, and preserve the sensory qualities of the final product. Ozonated water has been shown to be effective in reducing spoilage microorganisms without leaving harmful residues, making it a safer and environmentally friendly alternative to traditional chemical preservatives [17–19]. In the context of bakery products, the use of ozonated water has gained attention as a method to enhance dough quality and final product characteristics. Studies have demonstrated that ozonated water can improve gluten cross-linking, which in turn enhances the texture and crumb structure of baked goods. Moreover, ozonated water can also reduce the reliance on sodium-containing preservatives, which is particularly relevant in the development of low-sodium bakery products. This makes ozonated water a valuable tool in the ongoing efforts to produce healthier food options without compromising quality.

Comparatively, other methods such as the use of modified starches, gums, and alternative leavening agents have also been explored to achieve similar objectives. However, these methods often require extensive optimization to prevent negative impacts on sensory properties such as taste, aroma, and texture. Ozonated water, by contrast, offers a more straightforward and natural approach to achieving the desired product characteristics, with the added benefits of being residue-free and ecofriendly [20–22].

This study builds upon the existing literature by further exploring the application of ozonated water in the production of low-sodium cupcakes, aiming to optimize the technological parameters for achieving the best possible product quality [23, 24]. Ozonated water has the ability to oxidize sulfhydryl groups in proteins, which can lead to enhanced gluten cross-linking. This process improves the texture and crumb structure of low-sodium muffins [25–27]. However, careful optimization is required when using ozonated water in food products. Excessive oxidation could adversely affect sensory properties, such as flavor and mouthfeel, thus making it essential to fine-tune its application based on the specific characteristics of the food product.

## 2. Materials and Methods

This study involved the preparation of flour confectionery products (cupcakes) from ozonated water and finely ground whole-wheat flour of the “Naz” variety. The recipe for control and test cake samples is shown in Table 1.

The cake dough was prepared in the following modes: the initial temperature of the dough is 18°C, the final temperature is 24°C at an air pressure of 0.1–0.3 MPa and a rotation speed of the kneading body of 450–900 rpm, and the emulsion was whipped for 2 min. The total dough kneading time is 4 min.

The following indicators of the raw materials used and the resulting cupcakes were studied: moisture and dry matter content according to GOST 5900-73, mass fraction of fat according to GOST 5899-85, wetness according to GOST 10114-80, acidity and alkalinity according to GOST 5898-87, mass fraction of carbohydrates according to GOST 25832-89, mass fraction of protein according to GOST 10846-91, and others.

**Figure 1:**
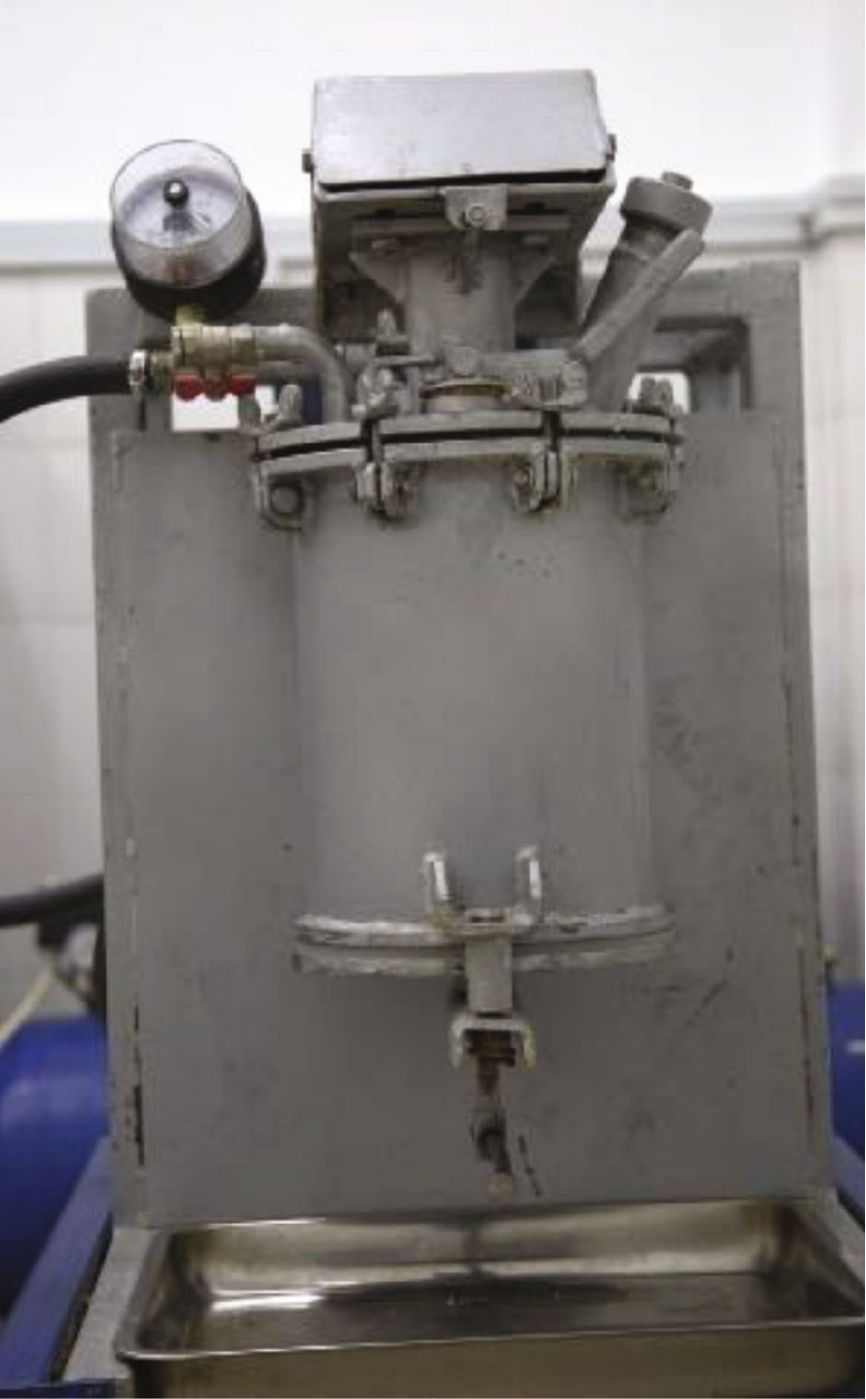
An image of the rapid dough-mixing apparatus.

The cupcake dough was produced by using an accelerated dough mixing machine as follows: the dough components were fed into the kneading body of a batch mixing machine through a loading hole in which a kneading element is installed, driven by an electric motor through a valve variator. At the end of loading, the kneading body of the dough mixing machine was hermetically sealed with a lid. The dough was kneaded for 3–5 min with a kneading body rotation speed of 5 s^−1^. Then, ionised cavitation (compressed) air was supplied to the kneading chamber at a pressure of 0.1 or 0.3 MPa, with the kneader shaft rotating at 450 or 900 rpm, and the dough was whipped for 10 min. When the recipe components were tipped, the test mass was saturated with air. The dough prepared in this way is a foamy mass with stable physicochemical characteristics. During unloading, the process of beating dough from whole-wheat flour was examined. The processing mode was regulated by varying the oscillation frequency and the magnitude of the centrifugal force field strength.  Figure 1 shows an image of the rapid dough-mixing apparatus with an ion–ozone cavitation unit [28].

The quality of the dough and flour confectionery products was assessed according to the following indicators: *y*_1_, dough moisture content (percent); and *у*_2_, dough alkalinity (degrees). The test deformations were designated as follows: *y*_3_, total deformation *H*_1_ (millimeter); *y*_4_, plastic deformation *Н*_2_ (millimeter); and *y*_5_, elastic deformation *Н*_3_ (millimeter). The physical indicators were designated as follows: *y*_6_, specific volume (grams per cubic meter); *y*_7_, dough temperature (degree Celsius); and *y*_8_, wettability of the finished product (percent). The chemical indicators were designated as follows: *y*_9_, the moisture content of the finished products (percent); *у*_10_, the alkalinity of the finished product (degrees); *y*_14_, the protein content of the finished product (percent); *у*_15_, the fat content of the finished product (percent); and *y*_16_, the carbohydrate content of the finished product (percent). The deformations of the finished product were designated as follows: *y*_11_, complete deformation of the finished products *H*_1_ (millimeter); *y*_12_, plastic deformation of the finished products *Н*_2_ (millimeter); and *у*_13_, elastic deformation of the finished product *Н*_3_ (millimeter). In addition, other chemical indicators were designated as follows: *y*_17_, the starch content of the finished product (percent); *у*_18_, the sugar content of the finished product (percent); *y*_19_, the fibre content of the finished product (percent); and *y*_20_, the ash content of the finished product (percent) [28].

The inclusion of ozone-treated water in muffin recipes reduces the microbial load without compromising the taste of the product. This approach allows for a longer shelf life and improved food safety in low-sodium muffins without the need for sodium-containing preservatives. Ozone is a highly reactive form of oxygen with a powerful oxidizing effect. When ozone is added to water, ozonized water is created, which can act as a powerful disinfectant in a variety of foods.

The antimicrobial properties of ozonized water are explained by its ability to destroy the cell walls of bacteria, fungi, and other pathogens, which subsequently leads to cell lysis and inactivation. The strong oxidative potential of ozonized water allows it to effectively reduce the number of microorganisms such as bacteria, molds, and yeasts in food products. This antimicrobial effect extends shelf life and ensures food safety.

However, one of the key challenges in using ozonized water is the potential loss of ozone to the environment, which can diminish its effectiveness as a bactericidal agent. To ensure maximum efficacy, it is crucial to optimize the conditions under which ozonized water is used. This includes controlling the concentration of ozone, minimizing its exposure to open air, and applying it under conditions that limit its dissipation. Careful attention to these factors will help maintain the bactericidal properties of ozonized water, ensuring that it provides the intended benefits in food safety and shelf life extension.

To reduce the influence of uncontrolled factors on the results, the experiments were randomized using tables of random numbers.  Table 2 presents the experimental conditions to produce cupcakes with fine whole-wheat “Naz” and ozonated water. A total of 16 cupcake samples were prepared.

## 3. Results and Discussion

### 3.1. Preparation of Cupcakes Using Fine Flour and Ozonated Water

Due to their high carbohydrate, fat, and protein contents, cupcakes are high-calorie, highly digestible products with a pleasant taste and attractive appearance. The technological process for preparing the cupcakes involved the following steps:

1. The margarine and granulated sugar were melted and then churned for 1–2 min. The eggs were added, and the mixture was churned for another 3–4 min until a fluffy foam formed. One to two minutes before the end, the flour was added, and the dough was beat to a certain consistency.
2. The dough was kneaded at an excess pressure (cavitation) of 0.1 or 0.3 MPa and at a kneading body speed of 450 or 900 rpm for 10 min.
3. The finished dough was placed in pregreased molds. The initial mass of the dough pieces was 120 g.
4. The cupcakes were baked at 180°C for 30 min without steam and moisture. The mass of each finished cupcake averaged 100 g.

**Figure 2:**
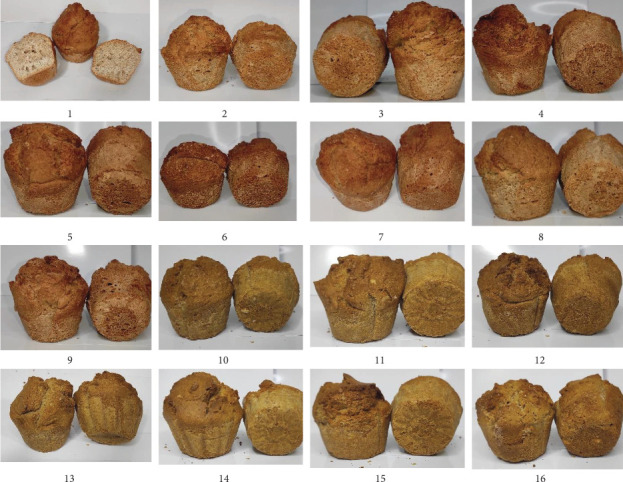
Images of the finished cupcakes.

The appearance of the 16 cupcake samples is shown in Figure 2.

For the sensory analysis, we employed a panel consisting of 10 trained experts who evaluated the cupcakes based on a set of predefined sensory attributes, including appearance, texture, flavor, and overall acceptability. The assessment was carried out using a 100% scale. The panelists were instructed to cleanse their palates with water between samples to avoid cross-sensory interference.

The sensory attributes were selected based on preliminary trials and relevant literature to capture the key characteristics influenced by the variation in ozone concentration, kneading speed, cavitation pressure, and flour percentage. Each cupcake sample was coded with a random three-digit number to ensure unbiased evaluation, and the samples were presented in a randomized order.

The data from the sensory evaluation were statistically analyzed using analysis of variance (ANOVA) to determine the significance of differences among the samples. Post hoc tests (Tukey's HSD) were used to identify specific differences between individual samples. The results of the organoleptic indicators of the cupcakes are shown in Table 3.

The most sensorially acceptable of the prepared cupcakes was No. 1, which exceeded the standard in taste, texture, and overall acceptability. A comparative analysis showed that No. 9 and No. 13 were closer in terms of indicators of No. 1.

### 3.2. Physical and Chemical Analyses of the Cupcakes

The physicochemical parameters of the 16 cupcake samples are shown in Table 4. The quality indicators of the dough and cupcakes were improved. Sample No. 1, No. 9, and No. 13 showed the best results.

Regression equations were obtained that adequately (according to the Fisher criterion) describe the influence of the technological factors of dough processing—*C*, ozone concentration (milligrams per cubic meter); *n*, the kneader shaft speed (revolutions per minute); *P*, cavitation pressure (megapascal); and *m*, the percentage of premium flour (percent)—on the quality indicators. Data processing and all necessary calculations were carried out using the PLAN sequential regression analysis program developed at the Odessa National Technological University. To determine the error variance (reproducibility), three parallel experiments were carried out. The regression coefficients were calculated by using matrices in natural dimensions, and, accordingly, the equations themselves were also obtained in natural dimensions. The general form of the regression equations for the four abovementioned factors (*C*, *n*, *P*, and *m*) is

(1) $$\begin{array}{c} y_{i}=b_{0}+b_{1}C+b_{2}n+b_{3}P+b_{4}m+b_{12}Cn+b_{13}CP+b_{14}Cm+b_{23}nP+b_{24}nm+b_{34}Pm, \end{array}$$

where *y*_*i*_ is the *i*-th indicator of the quality of the dough and cupcakes.

Table 5 provides the root mean square error (*S*_*e*_), the inadequacy (*S*_inad_), and the Fisher criterion (settlement [*F*_*s*_] and critical [*F*_*cr*_]). The obtained equations adequately describe the experimental data with a confidence level of *p* = 0.05.

The regression equations make it possible to predict the quality indicators of the dough and finished cupcakes obtained from them, depending on the values of *C*, *n*, *P*, and *m*. A brief analysis of the obtained regression equations revealed that of the 20 studied quality indicators, only the specific volume of the finished product (*y*_6_) does not depend on the dough processing factors. Only the dough temperature (*y*_7_) depends on one operating factor: as *P* increases, the dough temperature decreases. Three quality indicators of the finished product—alkalinity (*y*_10_), the carbohydrate content (*y*_16_), and starch (*y*_17_)—depend on two factors, namely *P* and *m*. Six quality indicators depend on three factors:

– The humidity of the dough (*y*_1_) depends on *C*, *n*, and *m*.
– The wetness (*y*_8_), humidity (*y*_9_), and fibre content (*y*_19_) of the finished product depend on *C*, *P*, and *m*.
– The fat content (*y*_15_) and sugar content (*y*_18_) of the finished product depend on *C*, *P*, and *n*.

The remaining nine indicators of the quality of the dough and finished product—alkalinity (*y*_2_), the total (*y*_3_), plastic (*y*_4_), and elastic deformation (*y*_5_) of the dough; the total (*y*_11_), plastic (*y*_12_), and elastic (*y*_13_) deformation of the finished product; and the protein (*y*_14_) and ash (*y*_20_) contents—depend on *C*, *n*, *P*, and *m*.

To optimize technological regimes for dough processing, the protein content in the finished cupcakes was chosen as the objective function:

(2) $$\begin{array}{c} y_{14}=8.7706-3.5350P-0.0100Cm+0.0030nP+0.1496Pm⟶\max \end{array}$$

**Figure 3:**
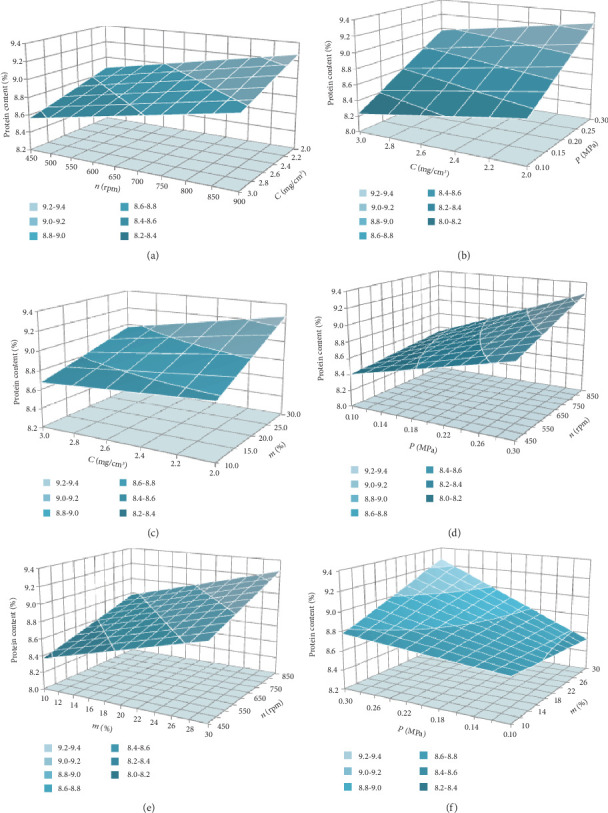
The response surfaces of the influence of the factors *C*, *n*, *P*, and *m* on the protein content of the finished flour confectionery products: (a) at *P* = 0.3 MPa and *m* = 30%; (b) at *n* = 900 rpm and *m* = 30%; (c) at *n* = 900 rpm and *P* = 0.3 MPa; (d) at *C* = 2 mg/cm^3^ and *m* = 30%; (e) at *C* = 2 mg/cm^3^ and *P* = 0.3 MPa; (f) at *C* = 2 mg/cm^3^ and *n* = 900 rpm.

Analysis of this equation revealed that the protein content of the finished cupcake is influenced by all four of the considered factors. *C* exerts its influence due to the significance of the pair interaction coefficient *b*_14_ and depends on *m*. *n* also affects the protein content nonlinearly, depending on *P*. In turn, *m* depends on *P*. *m* depends on *C* and *P*. Moreover, as *m* increases, to increase the protein content, *C* must be reduced, as indicated by the negative sign of the pair interaction coefficient *b*_14_. Thus, it is quite difficult to determine unambiguously analytically the influence of each factor on the protein content. More clearly, the nature of the joint mutual influence of the considered factors on the protein content in flour confectionery products (*y*_14_) can be determined by the response surfaces (Figure 3), constructed according to Equation (2).

Figure 3(a) shows the dependence of the protein content on *C* and *n* at a fixed *P* (0.3 MPa) and *m* (30%). The protein content increases slightly—by 0.30%—with a decrease in *C* and by 0.41% with an increase in *n*. The linear nature of the dependence is due to the absence in Equation (1) of the insignificant pair interaction coefficient *b*_12_.

The dependence of the protein content on *C* and *P*, shown in Figure 3(b), is similar. The protein content increases linearly by 0.71% with a decrease in *C* and by 0.30% with an increase in *P*. The linear dependence of the protein content on *C* and *P* is also explained by the absence of a significant pairwise interaction between them. The factors *n* and *m* in the considered dependence are 900 rpm and 30%, respectively.

At *n* = 900 rpm and *P* = 0.3 MPa (Figure 3(c)), there is a mutual influence of *C* and *m*. So, a decrease in *C* at *m* = 10% increases the protein content by 0.10%, and a decrease in *C* at *m* = 30% increases the protein content by 0.30%. Increasing *m* from 10% to 30% at *C* = 2.0 mg/cm^3^ increases the protein content by 0.5%; at *C* = 3.9 mg/cm^3^, the protein content increase is only 0.29%.

The dependence of *P* and *n* at *C* = 2 mg/cm^3^ and *m* = 30% has a similar character (Figure 3(d)). An increase in *P* and *n* leads to an increase in the protein content. An increase in *P* increases the protein content by 0.46% at *n* = 450 rpm and by 0.73% at *n* = 900 rpm. An increase in *n* also increases the protein content by 0.14% and 0.41%, respectively, at *P* = 0.1 and 0.3 MPa. The effect of the *P* and *n* interaction is not significant.

In Equation (1), the pairwise interaction between *n* and *m* has no effect, which makes their effect on the protein content straightforward at *C* = 2 mg/cm^3^ and *P* = 0.3 MPa (Figure 3(e)). An increase in *n*, regardless of *P*, increases the protein content by 0.41%, and an increase in *m*, also regardless of *P*, increases the protein content by 0.50%.

Figure 3(f) shows the nonlinear nature of the influence of *P* and *m* (at *C* = 2 mg/cm^3^ and *n* = 900 rpm) on the change in the protein content. An increase in *P* increases the protein content, but to varying degrees depending on *m*. So, with *m* = 10%, an increase in *P* increases the protein content by 0.14%, and with *m* = 30%, the protein content increases by 0.73%. The influence of *m* depending on *P* manifests itself in different ways. At the lowest pressure (*P* = 0.1 MPa), an increase in *m* reduces the protein content by 0.10%. On the contrary, at *P* = 0.3 MPa, the protein content increases by 0.50%. This is where the mutual influence of *P* and *m* manifests itself.

The production of flour confectionery products was optimized by considering the necessary quality indicators of the dough and finished cupcakes (Table 6).

The protein content was maximised in the range of changes in *C*, *n*, *P*, and *m* given in the experimental planning matrix (Table 6). The flour and dough processing modes using activated ion-ozonated water were optimized by using the nonlinear programming method. The following optimal technological modes for dough processing were obtained:

– *C* = 2 mg/cm^3^,
– *n* = 900 rpm,
– *P* = 0.3 MPa,
– *m* = 30%.

Under these optimal dough processing conditions, the target function (protein content in the finished flour confectionery products) is 9.27%. The values of other dough and finished cupcake quality indicators under optimal dough processing conditions are given in Table 7.

Overall, the use of ozonated water for preparing the dough and its processing according to optimal technological conditions made it possible to maintain the studied dough quality indicators within acceptable limits. It also ensured the maximum protein content in the finished cupcakes. The results are explained with the data presented in Tables 5, 6, and 7 and Figures 2 and 3. Hence, a technology has been developed to prepare flour confectionery products with an accelerated production cycle based on whole-wheat flour, ozonated water, and cavitation treatment of the dough.

Considering the verified multifactorial design of the experiments based on three criteria, three samples showed the best performance: No. 1, No. 9, and No. 13. In these samples, differentiated preparation and processing regimes were applied in accordance with the plans of full four factorial experiments (Type 24). The best quality samples were prepared and processed in the following modes:

– Sample No. 1: *C* = 3 mg/cm^3^, *n* = 900 rpm, *P* = 0.3 MPa, and *m* = 30%,
– Sample No. 9: *C* = 3 mg/cm^3^, *n* = 900 rpm, *P* = 0.3 MPa, and *m* = 10%,
– Sample No. 13: *C* = 3 mg/cm^3^, *n* = 900 rpm, *P* = 0.3 MPa, and *m* = 10%.

Comparison of the quality indicators of the 16 cupcake samples identified the following rational preparation parameters: *C* = 3 mg/cm^3^, *n* = 900 rpm, and *m* = 10% of the total mass.

The developed linear and nonlinear models based on the target function for the protein content of the finished cupcakes made it possible to establish the optimal mode of cavitation technology for dough preparation. Sample No. 1 is the optimal option for preparing the dough in an accelerated way. Kneading dough under pressure and using activated ozonated water can significantly reduce the kneading time by up to 20% and improve the structure of the dough. This approach also improves the physicochemical and organoleptic characteristics of the cupcake, increasing the specific volume and porosity of the crumb. The disadvantage of this method is that the recipe to prepare the flour confectionary products includes either butter or margarine, the latter of which is a product of chemical hydrogenation of vegetable oils and contains trans fatty acids. The presence of a large number of trans fatty acids in food disrupts the metabolism of fatty acids in the human body and contributes to the deficiency of many important compounds, which can lead to an increase in the incidence of atherosclerosis and other diseases. Therefore, for cooking, it is necessary to use natural butter that does not contain trans fatty acids.

Many leading scientists in the world have obtained different assortments of flour confectionery products, obtained by accelerated preparation methods, with increased nutritional and biological value [29–32]. Scientists Magomedov et al. developed technologies for aerated flour confectionery products of increased nutritional value using triticale flour, and new methods of aeration were used that increased nutritional value [33]. However, the accelerated ozone cavitation technology we used in the study for preparing muffins from whole-ground flour without adding baking powder helps not only to ensure high safety but also to increase production efficiency and stabilize the quality of the finished product. This technology allows manufacturers to obtain high-quality and safe flour confectionery products using an accelerated method.

This work provides a new direction to improve public health through the creation of food products based on environmentally friendly technologies using whole-wheat flour. The results of the work should contribute significantly to the development of science and technology in the food industry. The expected social effect will be the use of ozonated water, which has many useful properties (bactericidal, redox, etc.), and the use of cavitation treatment of dough in the production of flour confectionery products. This approach would create environmentally friendly products, which is very important for consumers, given that many people live in environmentally unfavourable regions and work in contact with harmful chemical and biological environmental factors. The economic effect will be achieved by reducing the duration of the production process.

## 4. Conclusion

The application of ozonated water in the preparation of cupcakes was carefully evaluated to assess its impact on sensory quality and residual taste. The oxidation of sulfhydryl groups in proteins, induced by ozonated water, contributed to enhanced gluten cross-linking, which in turn improved the overall texture and crumb structure of the cupcakes. Importantly, this method did not negatively affect the taste profile of the product. The sensory analysis indicated that the use of ozonated water preserved the favorable sensory properties, and no off-flavors or undesirable residual tastes were detected. These findings suggest that ozonated water can be effectively used in low-sodium formulations to maintain product quality without compromising sensory attributes.

This research developed a recipe and technological regime for the preparation of flour confectionery products, including cupcakes, made from finely ground whole-wheat flour and ozonated water. The basic principles of mechanical leavening were identified, and low-sodium cupcakes were developed without compromising the texture, volume, or other sensory characteristics. Ozonated water oxidises sulfhydryl groups in proteins, helping to enhance gluten cross-linking and to improve the overall texture and structure of the muffin crumb, moreover, eliminating baking soda and sodium from the recipe. Sample No. 1 had the optimal physical and chemical characteristics, with *C*3mg/cm^3^, *n* = 900 rpm, *P* = 0.3 MPa, and *m* = 30%.

To determine the shelf life of the cupcakes prepared with ozonated water, a series of storage tests were conducted under controlled conditions (25°C, 60% relative humidity). The samples were observed over a period of 7, 14, and 21 days to assess their physical appearance, moisture content, and microbial load.

The syneresis test was conducted to evaluate the stability of the cupcake's texture over time. This test measured the extent of liquid separation in the cupcake matrix, which is critical for understanding the product's quality during storage. Syneresis was measured using a gravimetric method where any liquid released from the cupcake was quantified at specific intervals during the storage period.

The cupcakes exhibited minimal syneresis throughout the storage period, indicating that the ozonated water contributed to a stable product structure. Additionally, the microbial analysis showed that the use of ozonated water effectively inhibited the growth of spoilage microorganisms, thereby extending the shelf life of the product to at least 21 days without the use of chemical preservatives.

## Figures and Tables

**Table 1:** Recipe for control and test cake samples.

**No.**	**Raw material**	**For control samples of cupcakes**	**For studied cake samples**
1	Finely ground whole-wheat flour (g)	600	—
2	High-grade flour (g)	—	600
3	Butter (g)	300	300
4	Chicken egg (g)	325	325
5	Sugar (g)	200	200
6	Ozone water (mL)	120	120
	Yield of dough pieces (g)	1545	1545
	Yield of finished products (g)	1125	1175

**Table 2:** Experimental conditions using finely ground whole-wheat “Naz” flour and ozonated water to prepare cupcakes.

**No.**	**Factors**			
	**Ozone concentration (** **C** **, mg/cm** ^**3**^ **)**	**Kneader shaft speed (** **n** **, rpm)**	**Cavitation pressure** **(** **P** **, MPa)**	**Percentage of premium flour (** **m** **, %)**
1	3	900	0.3	30
2	2	900	0.3	30
3	3	450	0.3	30
4	2	450	0.3	30
5	3	900	0.1	30
6	2	900	0.1	30
7	3	450	0.1	30
8	2	450	0.1	30
9	3	900	0.3	10
10	2	900	0.3	10
11	3	450	0.3	10
12	2	450	0.3	10
13	3	900	0.1	10
14	2	900	0.1	10
15	3	450	0.1	10
16	2	450	0.1	10

**Table 3:** The results of the organoleptic indicators of the cupcakes.

**Sample no.**	**Appearance (%)**	**Taste (%)**	**Texture (%)**	**Aroma (%)**	**Over acceptability (%)**	**Average value (%)**
Control	75	68	60	72	75	70
1	73	65	56	68	72	67
2	65	52	50	65	68	60
3	70	63	53	67	70	65
4	64	55	49	62	65	59
5	64	56	51	63	61	59
6	66	50	52	68	63	60
7	60	63	55	63	56	59
8	69	57	51	59	65	60
9	64	58	56	48	58	57
10	70	50	51	59	60	58
11	56	60	54	56	60	57
12	52	48	50	55	62	53
13	71	63	56	68	70	66
14	62	55	53	66	69	61
15	66	54	53	68	70	62
16	65	60	55	68	69	63

**Table 4:** The quality indicators of the dough and finished cupcakes made from finely ground whole-wheat “Naz” flour and ozonated water.

**No.**	**Physical and chemical indicators**																			
	**y**_1_ ** (%)**	**y**_2_ ** (°)**	**y**_3_ ** (mm)**	**y**_4_ ** (mm)**	**y**_5_ ** (mm)**	**y**_6_ ** (g/сm ** ^**3**^ **)**	**y**_7_ ** (°С)**	**y**_8_ ** (%)**	**y**_9_ ** (%)**	**y**_10_ ** (°)**	**y**_11_ ** (mm)**	**y**_12_ ** (mm)**	**y**_13_ ** (mm)**	**y**_14_ ** (%)**	**y**_15_ ** (%)**	**y**_16_ ** (%)**	**y**_17_ ** (%)**	**y**_18_ ** (%)**	**y**_19_ ** (%)**	**y**_20_ ** (%)**
1	29.43	1.82	13	12.6	0.64	199	18.5	113.34	21.8	0.77	9.76	6.5	3.26	8.73	19.98	46.14	43.18	3.47	7.12	0.88
2	25	2.16	17.8	17	0.8	203	18.8	134.51	30.43	1.35	6.3	3.9	2.4	9.19	21.38	41.96	38.21	3.19	6.29	0.9
3	32.71	2.76	14.3	13.5	0.8	200	17.9	121.42	32.17	1.59	11.6	7.6	4	8.56	20.41	37.88	34.07	2.27	8.41	0.62
4	29.94	1.99	18.25	17.13	1.12	203	17.7	159.72	22.53	1.03	3.6	2.1	1.5	9.19	23.79	50.25	47.51	2.15	7.38	0.92
5	29.76	1.78	8.25	7.13	1.12	202	19.1	144.1	26.62	0.77	6.4	4.3	2.1	8.68	23.9	63.04	59.62	3.19	9.51	0.97
6	29.1	1.75	11.6	10.6	1.0	200	18.7	198.91	22.12	0.58	5.35	3.3	2.05	8.76	18.66	50.25	47.12	2.05	6.79	1.05
7	29.6	1.93	9.2	8.23	1.13	200	19.1	147.56	24.96	0.39	5.8	3.99	1.81	7.85	21.98	51.47	48.56	3.62	7.68	0.86
8	28.81	1.53	14.9	13.9	1.0	204	19.3	143.06	21.2	0.6	3.25	1.9	1.35	7.93	20.92	49.78	45.47	2.71	8.81	0.99
9	28.74	1.37	10.75	9.9	0.85	202	18.2	133.08	15.6	1.0	3.1	1.78	1.32	8.57	21.92	53.57	50.31	2.83	9.11	0.75
10	29.31	1.89	11.1	10.25	0.85	200	17.1	101.01	24	1.2	3.25	2.1	1.11	9.26	22.4	45.45	41.83	3.01	9.23	0.71
11	26.78	1.63	7.8	6.9	0.9	202	18	115.3	20.6	1.0	2.0	1.5	1.1	8.45	20.32	59.09	55.91	3.11	8.39	1.12
12	29.85	1.66	10.75	9.75	1.0	201	19.2	111.13	25.6	0.9	2.26	0.48	1.17	7.78	19.44	60.77	57.97	2.38	7.58	0.71
13	27.71	1.83	10.75	10	0.75	203	19	139.58	22.2	1.63	3.3	2.2	1.1	7.91	18.57	56.01	52.48	3.41	6.83	1.13
14	30.19	2.94	11.8	11.2	0.6	203	19.1	120.54	23.2	1.6	5.25	2.47	1.65	8.38	19.63	62.5	59.63	2.56	8.39	1.13
15	28.43	2.83	11.35	10.5	0.8	201	17.5	125.9	18	1.8	2.3	1.25	1.05	9.17	21.81	58.94	55.05	2.68	9.19	1.18
16	26.84	2.54	9.88	9.11	0.9	200	17.8	96.62	26.84	2.3	2.2	1.3	0.9	8.66	22.39	57.83	54.07	3.71	7.97	1.24

**Table 5:** Regression equations and statistical characteristics of the dependence of the quality indicators of the dough and finished cupcakes on the four factors that influence them (*C*, *n*, *v*, *P*, and *m*).

**Regression equations**	**Standard deviation**		**Fisher's criterion**	
	**S**_**e**_	**S**_**i** **n** **a** **d**_	**F**_**s**_	**F**_**c** **r**_
*y*_1_ = 28.0211 + 0.0482*Сm* − 0.00011*nm*	0.57	1.58	7.71	19.42
*y*_2_ = 3.960 + 0.00278*n* − 7.8187*Р* − 0.1653*m* − 0.00126*С* + 0.0370*Сm* + 0.3331*Рm*	0.11	0.22	4.03	19.38
*y*_3_ = 12.7700 − 31.4042*Р* + 0.3652*m* − 0.1407*Сm* + 0.01917*nР* − 0.00023*nm* + 1.4237*Рm*	0.51	0.97	3.62	19.38
*y*_4_ = 12.3462 − 34.3750*Р* + 0.3315*m* − 0.1363*Сm* + 0.02093*nР* − 0.00023*nm* + 1.5237*Рm*	0.55	0.93	2.89	19.38
*y*_5_ = 0.1821*С* + 5.2025*Р* + 0.02394*m* − 1.0883*СР* − 0.00133*nР* − 0.08977*Рm*	0.045	0.086	3.65	19.39
*y*_6_ = 201.4375	2.80	1.50	3.47	3.68
*y*_7_ = 18.9625 − 2.6250*Р*	0.20	0.67	11.10	19.42
*y*_8_ = 47.8364*С* − 78.2634*Р* + 7.6948*m* − 2.5254*Сm*	6.56	15.72	5.74	19.41
*y*_9_ = 18.8314 + 44.3095*Р* − 15.8263*СР* + 0.07674*Сm*	1.17	3.86	10.90	19.41
*y*_10_ = 3.2119 − 7.5562*Р* − 0.09756*m* + 0.3519*Рm*	0.17	0.26	2.37	19.41
*y*_11_ = 1.5862 + 0.01760*n* − 34.4458*Р* − 0.4677*m* − 0.00596*Сn* + 9.3333*СР* + 0.1936*Сm* + 0.8062*Рm*	0.24	0.98	16.56	19.37
*y*_12_ = 0.01095*n* − 22.2064*Р* − 0.2761*m* − 0.00359*Сn* + 6.7792*С* + 0.1275*Сm* + 0.4412*Рm*	0.14	0.52	13.83	19.38
*y*_13_ = 0.00590*n* − 8.2779*Р* − 0.1045*m* − 0.00174*Сn* + 3.6792*СР* + 0.04604*Сm* − 0.00463*nР* + 0.2325*Рm*	0.084	0.331	15.48	19.37
*y*_14_ = 8.771 − 3.535*Р* − 0.010*Сm* + 0.003*nР* + 0.150*Рm*	0.11	0.47	18.05	19.40
*y*_15_ = 21.8725 + 1.4807*С* − 0.00685*n* − 7.2286*СР* + 0.02782*nР*	0.43	1.66	14.91	19.40
*y*_16_ = 59.9778 − 1.7924*Рm*	1.94	5.18	7.13	19.42
*y*_17_ = 56.5377 − 1.7752*Рm*	1.87	5.45	8.50	19.42
*y*_18_ = 3.8419 + 0.4387*С* − 0.00255*n* − 10.3625*Р* + 0.01331*nР*	0.12	0.40	11.17	19.40
*y*_19_ = 7.1850 + 6.9525*Р* + 0.02130*Сm* − 0.3995*Рm*	0.21	0.91	18.83	19.41
*y*_20_ = 1.6625 − 0.00056*n* − 3.0875*Р* + 0.7500*СР* − 0.0090*Сm* + 0.00003*nm*	0.046	0.129	6.39	19.39

**Table 6:** The limits of the quality indicators of the dough and finished cupcakes.

**Min**		**Equation**		**Max**
23.5	≤	*y*_1_ = 28.0211 + 0.0482*Cm* − 0.00011*nm* (%)	≤	33.5
1.2	≤	*y*_2_ = 3.960 + 0.00278*n* − 7.8187*P* − 0.1653*m* − 0.00126*C* + 0.0370*Cm* + 0.3331*Pm* (°)	≤	3.0
7.2	≤	*y*_3_ = 12.770 − 31.4042*P* + 0.3652*m* − 0.1407*Cm* + 0.01917*nP* − 0.00023*nm* + 1.4237*Pm* (mm)	≤	18.75
6.5	≤	*y*_4_ = 12.3462 − 34.3750*P* + 0.3315*m* − 0.1363*Cm* + 0.02093*nP* − 0.00023*nm* + 1.5237*Pm* (mm)	≤	17.5
0.2	≤	*y*_5_ = 0.1821*C* + 5.2025*P* + 0.02394*m* − 1.0883*CP* − 0.00133*nP* − 0.08977*Pm* (mm)	≤	1.15
196	≤	*y*_6_ = 201.44 (g/сm^3^)	≤	206
16.9	≤	*y*_7_ = 18.9625 − 2.6250*P* (°С)	≤	19.5
95.0	≤	*y*_8_ = 47.8364*C* − 78.2634*P* + 7.6948*m* − 2.5254*Cm* (%)	≤	200.0
15.0	≤	*y*_9_ = 18.8314 + 44.3095*P* − 15.8263*CP* + 0.07674*Cm* (%)	≤	32.6
0.2	≤	*y*_10_ = 3.2119 − 7.5562*P* − 0.09756*m* + 0.3519*Pm* (°)	≤	2.5
2.0	≤	*y*_11_ = 1.5862 + 0.01760*n* − 34.4458*P* − 0.00596*Cn* + 9.3333*CP* + 0.1936*Cm* + 0.8062*Pm* (mm)	≤	10.0
1.5	≤	*y*_12_ = 0.01095*n* − 22.2064*P* − 0.2761*m* − 0.00359*Cn* + 6.7792*C* + 0.1275*Cm* + 0.4412*Pm* (mm)	≤	8.0
0.9	≤	*y*_13_ = 0.00590*n* − 8.2779*P* − 0.1045*m* − 0.00174*Cn* + 3.6792*CP* + 0.04604*Cm* − 0.00463*nP* + 0.2325*Pm* (mm)	≤	4.5
7.5	≤	*y*_14_ = 8.771 − 3.535*P* − 0.010*Cm* + 0.003*nP* + 0.150*Pm* (%)	≤	10.25
17.5	≤	*y*_15_ = 21.8725 + 1.4807*C* − 0.00685*n* − 7.2286*CP* + 0.02782*nP* (%)	≤	24.5
36.0	≤	*y*_16_ = 59.9778 − 1.7924*Pm* (%)	≤	65.0
33.07	≤	*y*_17_ = 56.5377 − 1.7752*Pm* (%)	≤	61.60
2.0	≤	*y*_18_ = 3.8419 + 0.4387*C* − 0.00255*n* − 10.3625*P* + 0.01331*nP* (%)	≤	4.0
5.5	≤	*y*_19_ = 7.1850 + 6.9525*P* + 0.02130*Cm* − 0.3995*Pm* (%)	≤	10.51
0.55	≤	*y*_20_ = 1.6625 − 0.00056*n* − 3.0875*P* + 0.7500*CP* − 0.0090*Cm* + 0.00003*nm* (%)	≤	2.20

**Table 7:** The quality indicator values for the dough and finished cupcakes prepared with finely milled whole-wheat “Naz” flour and ozonated water.

**Indicators**	**Min**		**Opt**		**Max**
*у*_1_—Dough moisture content (%)	23.5	≤	27.83	≤	33.5
*у*_2_—Dough alkalinity (°)	1.2	≤	2.11	≤	3.0
*у*_3_—Total deformation *Н*_1_ (mm)	7.2	≤	17.71	≤	18.75
*у*_4_—Plastic deformation *Н*_2_ (mm)	6.5	≤	16.96	≤	17.5
*у*_5_—Elastic deformation *Н*_3_ (mm)	0.2	≤	0.82	≤	1.15
*у*_6_—Specific volume (v/m^3^)	196	≤	201.4	≤	206
*у*_7_—Dough temperature (°С)	16.9	≤	18.18	≤	19.5
*у*_8_—Wetness of finished products (%)	95.0	≤	151.5	≤	200.0
*у*_9_—Humidity of finished products (%)	15.0	≤	27.23	≤	32.6
*у*_10_—Alkalinity of finished products (°)	0.2	≤	1.19	≤	2.5
*у*_11_—Total deformation of finished products *Н*_1_ (mm)	2.0	≤	6.80	≤	10.0
*у*_12_—Plastic deformation of finished products *Н*_2_ (mm)	1.5	≤	4.14	≤	8.0
*у*_13_—Elastic deformation of finished products *Н*_3_ (mm)	0.9	≤	2.36	≤	4.5
*у*_14_—Protein of finished products (%)	7.5	≤	9.27	≤	10.25
*у*_15_—Fats of finished products (%)	17.5	≤	21.85	≤	24.5
*у*_16_—Carbohydrates of finished products (%)	36.0	≤	43.85	≤	65.0
*у*_17_—Starch of finished products (%)	33.07	≤	40.56	≤	61.60
*у*_18_—Sugar finished products (%)	2.0	≤	2.91	≤	4.0
*у*_19_—Fibre of finished products (%)	5.5	≤	6.95	≤	10.51
*у*_20_—Ash of finished products (%)	0.55	≤	0.85	≤	2.20

## Data Availability

Data will be made available on request.

## Appendix A: Recommendations

Based on the results, this topic is relevant to the field of food science and technology to produce a new range of flour confectionery products with increased nutritional value. The developed highly efficient accelerated technology to produce flour confectionery products, including cupcakes, from finely ground whole-wheat flour should allow manufacturers to intensify biotechnological production processes.
